# Health-related quality of life of unemployed individuals diagnosed with a mental illness: a cross-sectional study

**DOI:** 10.1007/s11136-025-04051-5

**Published:** 2025-09-03

**Authors:** A. P. Liegert, F. S. Hussenoeder, M. Koschig, M. Alberti, L. Bieler, C. Rohde, K. Stengler, S. G. Riedel-Heller, I. Conrad

**Affiliations:** 1 Institute of Social Medicine, Occupational Health and Public Health, Leipzig University, Ph.-Rosenthal-Str. 55, 04103 Leipzig, Germany; 2 Clinic for Psychiatry, Psychosomatics and Psychotherapy, Helios Park-Klinikum, Leipzig, Germany

**Keywords:** Unemployment, Work, Health-related quality of life, SF-12, Mental health

## Abstract

**Background:**

Unemployment is a recognized risk factor for impaired physical and mental health, and numerous studies have shown that unemployed people often report a reduced health-related quality of life (HRQoL). The concurrent effects of having a mental illness and being unemployed reinforce one another, leading to chronic symptoms and reduced employability. This study examined the relationships between unemployment, HRQoL and other work- and health-related factors in unemployed individuals with mental illnesses.

**Methods:**

We analyzed cross-sectional data from unemployed adults participating in the “Leipzig—Individual Placement and Support for people with mental illnesses” (LIPSY) project. All participants met ICD-10 criteria for at least one mental disorder. HRQoL was assessed with the 12-Item Short-Form Survey (SF-12), yielding a Physical Component Summary (PCS) and Mental Component Summary (MCS). Two multiple regression analyses (outcomes: PCS, MCS) included age, gender, education, partnership status, social network size, unemployment duration (≤ 24 vs 24 months), work ability score (WAS), and depression, anxiety, and somatization (Mini-Symptom Checklist, Mini-SCL) as predictors.

**Results:**

Our sample included 452 unemployed participants with mental illnesses with an average age of 35.5 years, 50.4% were female. Higher age and somatization were negatively associated with PCS, while female gender, work ability, and anxiety showed positive associations. MCS was negatively associated with higher education, depression, and anxiety, and positively with work ability.

**Conclusion:**

Our findings highlight the complex associations between demographic, psychological, and work-related factors and HRQoL. Supportive measures for unemployed individuals could have a dual impact by improving both HRQoL and employability.

## Abstract (plain language summary)

1. **Why is this study needed?**
  Job loss is often a harmful experience, even more so for people who already have a mental illness. We wanted to learn more about the factors that hurt or help health-related quality of life so that future support programs could be better implemented.
2. **What is the key problem/issue/question this manuscript addresses?**
  How are age, education, mental-health symptoms, and perceived work ability linked to physical and mental well-being in unemployed people with mental illnesses?
3. **What is the main point of your study?**
  We want to investigate what factors most affect the well-being of our study participants so that we can help to improve the care and support of unemployed people struggling with mental illness.
4. **What are your main results and what do they mean?**
  Overall, people in our study felt much less healthy than the general population. Specifically, depression, anxiety, somatic complaints, and low work ability were strongly tied to poorer quality of life. Older people and those with more bodily complaints felt worse physically, but not necessarily mentally. Women, people who felt more able to work, and some with anxiety felt better physically. People with more education, depression, or anxiety felt worse mentally. Feeling able to work helped both physical and mental well-being. This suggests that helping people feel ready to work and supporting their health can improve how they feel and help them get back to work.

## Introduction

Health-related quality of life (HRQoL) refers to how individuals perceive and evaluate their physical and mental health in relation to their everyday life and functioning [1]. Multiple studies have shown that unemployed people have consistently reported a lower HRQoL compared to employed reference groups [2–5]. Persistent health impairments may lead to symptoms worsening and increase the risk of chronic illness, making a return to work increasingly difficult [6, 7]. Recent German data indicate that 52% of unemployed individuals meet criteria for at least one psychiatric disorder [8].

Our study focuses on the HRQoL of unemployed individuals with mental illnesses and provides important information on the factors connected to perceived health within this highly vulnerable, under-researched group. Research has shown that factors such as psychiatric symptoms are related to lower overall HRQoL [9, 10]. Further, long-term unemployed individuals with elevated levels of depression and anxiety perceived their own health as poor [3].

Nevertheless, research on HRQoL in unemployed people with diagnosed mental disorders remains sparse. Available evidence suggests that demographic and psychosocial factors may shape HRQoL in this group. For example, longer durations of unemployment correlates with poorer HRQoL [11]. With regards to gender and age, a Swedish study found negative effects for men and for younger unemployed individuals [4]. Social networks may have a positive effect on HRQoL [12, 13] and higher education and socioeconomic status also appear to be protective [5]. In addition to mental illness, several physical conditions have been found to further contribute to declines in HRQoL. In patients with cardiovascular disease or chronic pancreatitis, being unemployed is linked to substantially worse HRQoL [14, 15]. Self-assessed work ability may serve as a protective factor, predicting better HRQoL and facilitating labor market reintegration [16].

To examine HRQoL in a differentiated way, our study conceptualizes HRQoL as comprising two distinct components: the Physical Component Summary (PCS) and the Mental Component Summary (MCS) [1]. The PCS reflects physical functionality and the extent to which physical health problems limit everyday activities, whereas the MCS assesses the impact of psychological distress on well-being and social functioning [17].

Previous research has shown that both PCS and MCS are lower in unemployed individuals compared to the general population [3]. Furthermore, studies have demonstrated that among unemployed individuals, mental health perception is generally more adversely affected than physical health perception [2, 18].

The Mental Component Score (MCS) has been found to be lower in women, individuals with higher depression and anxiety scores, and those with low levels of physical activity. In contrast, a lower Physical Component Score (PCS) is associated with older age, higher body mass index (BMI), and elevated depression and anxiety scores [3].

Based on this literature overview and the limited number of comparable studies in this population, the present study seeks to identify factors that impact individuals who are both unemployed and diagnosed with mental health disorders by pursuing two specific objectives: (1) to contextualize the PCS and MCS by comparing them with findings from the general population and from existing studies on similar target groups and (2) to investigate, within an explanatory framework, which sociodemographic, clinical, and work-related factors are independently associated with PCS and MCS. By identifying both risks and resources, our findings contribute to the development of tailored treatment programs and preventive strategies for this highly vulnerable population. Therefore, we seek to explore these associations in the context of the German healthcare and social support systems based on our sample data.

## Methods

### Study design

The sample for this study is comprised of participants from “Leipzig Individual Placement and Support for Mentally Ill People (LIPSY)”, a project which aims to support individuals with mental illness in their reintegration into the labor market. Inclusion criteria were: (1) receiving means-tested unemployment benefits (German: ALG 2/ Bürgergeld), (2) at least one diagnosed psychiatric disorder according to the International Statistical Classification of Diseases and Related Health Problems, 10th Revision (ICD-10, F codes), and (3) sufficient German language skills. Individuals not meeting these criteria were excluded from participation [19, 20].

Data were collected via self-assessment questionnaires at four predefined time points and included sociodemographic, work-related, and health-related variables. During the project period, participants received targeted psychosocial interventions and vocational training tailored to their individual needs [19].

A total of 600 participants were initially enrolled in the study at a Job Center in Germany. For the present cross-sectional analysis, baseline data were used. After excluding cases with missing values on any of the variables included in the analysis, the final sample comprised 452 participants. We compared sex, age, and education between included and excluded individuals and found no significant differences. This supports the assumption of missing completely at random (MCAR) and justifies complete case analysis [21].

### Ethics

The survey was conducted in accordance with the Helsinki Declaration of 1975 (revised in 2008) and was approved by the Ethics Committee of the Medical Faculty of the University of Leipzig. Written informed consent was obtained from all participants.

### Measures

*Sociodemographic variables.* All participants were asked to provide information on age, gender, education, cohabitation with a partner (yes/no) and duration of unemployment in months. The information on education was divided into four categories: (1) no school diploma, (2) lower level (secondary school qualification), (3) medium level (intermediate or 10th grade polytechnic secondary school certificate), and (4) higher level (advanced technical college qualification, technical secondary school leaving certificate, general/specialized higher education entrance qualification/abitur). Unemployment duration was dichotomized as “up to 24 months” versus “more than 24 months” to distinguish unemployment from very long-term unemployment [22].

*Social network size.* The brief version of the Lubben Social Network Scale (LSNS-6) was employed to quantify the size of participants' social networks [23]. The scale contains 6 items with a 6-point scale referring to social connections with family members, friends and neighbors. Total scores range from 0 to 30, with higher values indicating larger social networks. Internal consistency in the current sample was acceptable (Cronbach’s α = .79).

*Work Ability Score*. Work ability was assessed using a single item from the Work Ability Index (WAI) [24], commonly referred to as the Work Ability Score (WAS) [25, 26]. Participants rated their current work ability on a scale from 0 = “completely unable to work” to 10 = “best working ability ever experienced”. 

*Psychological Symptoms.* Depressive, anxiety and somatic symptoms were measured using the Mini-Symptom Checklist (Mini-SCL), with each subscale consisting of six items. Items were rated on a 5-point scale from 0 = “not at all” to 4 = “very strongly” [27] with subscale scores ranging between 0 and 24. Internal consistency was acceptable for the depression (α = .80) and somatization (α = .80) subscales, and excellent for the anxiety subscale (α = .92).

*Health-related quality of life.* The Short Form-12 Health Survey (SF-12), a brief form of the SF-36, employs 12 questions to subjectively assess HRQoL [28], resulting in two final scores: the Physical Component Summary (PCS) and the Mental Component Summary (MCS). In both cases, higher scores indicate better self-perceived physical or mental health status. The questionnaire is frequently used in disease-specific clinical and therapeutic settings but is also suitable for population studies [29, 30]. Both scores were calculated via the algorithm provided in the SF-12 manual [1].

### Statistical analyses

All statistical data analyses were conducted using IBM SPSS Version 27. Two multiple linear regression models were computed to examine predictors of HRQoL. Predictor variables included age, gender, educational level, cohabitation status, social network size, duration of unemployment, self-assessed work ability, and symptom levels of depression, anxiety, and somatization. In the first model, the Mental Component Score (MCS) served as the dependent variable; in the second model, the Physical Component Score (PCS) was used as the outcome. Prior to model estimation, multicollinearity was assessed using both the correlation matrix and variance inflation factors (VIF). All VIF values were below 3.3, indicating no critical multicollinearity among predictors [31].

## Results

Our final sample included 452 unemployed participants who had been diagnosed with at least one psychiatric disorder. The mean age was 35.5 years (*SD* = 11.0) and 50.4% of the participants were female. The median duration of unemployment was 36.0 months (*IQR* = 16.25–72.00). Table 1 provides an overview of the general characteristics of the sample.

**Table 1:** General Characteristics of the Study Population

	Total Group (*N *= 452)
Age (years)	35.53 (11.01)
Gender (“female”)	228 (50.4%)
Education	
No school leaving certificate	41 (9.1%)
Low (secondary general school)	104 (23.0%)
Medium (intermediate/polytechnic secondary school)	188 (41.6%)
High (technical secondary school, university entrance qualification)	119 (26.3%)
Living with a partner (“yes”)	75 (16.6%)
Social network size (LSNS-6; range: 0–30)	10.38 (5.71)
Unemployment	
Duration in months (median, IQR)	36.00 (16.25–72.00)
Longterm unemployed (> 24 months)	263 (58.2%)
Work Ability Score (from WAI; range: 0–10)	3.31 (2.34)
Mini-Symptom Checklist (Mini-SCL)	
Depression (range: 0–24)	10.32 (5.22)
Anxiety (range: 0–24)	7.56 (5.15)
Somatization (range: 0–24)	5.63 (4.65)
Short Form Health Survey (SF-12)	
PCS (range: 0–100)	43.29 (10.51)
MCS (range: 0–100)	30.86 (9.13)

For continuous variables the mean (standard deviation) is presented with the exception of unemployment duration (median); categorical variables are displayed as numbers (percentages)

Mean scores for HRQoL were below the normative mean of 50.0 in the German general population [1] for both PCS (*M* = 43.29, *SD* = 10.51) and MCS (*M* = 30.86, *SD* = 9.13).

Table 2 displays the results of the multiple linear regression analyses. For PCS, significant negative associations were observed with age (*β* = -.22) and somatization (*β* = -.44), while significant positive associations were found with gender (*β* = .10), work ability (*β* = .28), and anxiety (*β* = .13).

**Table 2:** Associations between predictor variables and health-related quality of life (SF-12: PCS and MCS) (standardized regression coefficients; *N* = 452)

	SF-12 PCS	SF-12 MCS
Age	− 0.22***	0.01
Gender (“female”)^1^	0.10*	0.02
Education (“no certification“)		
low	− 0.02	0.00
moderate	− 0.00	− 0.11
high	0.06	− 0.19**
Living with partner (“no”)	− 0.04	− 0.02
Social network size	0.05	− 0.02
Longterm unemployment (“<=24 months“)	− 0.03	0.04
Work Ability Score	0.28***	0.14***
Depression	0.08	− 0.50***
Anxiety	0.13*	− 0.16***
Somatization	− 0.44***	0.05
R^2^	0.36	0.41

*p ≤ 0.05; **p ≤ 0.01; ***p ≤ 0.001. Significant coefficients are highlighted in bold

^1^The category coded as “0” (= reference category) is presented in parentheses

For MCS, higher education (*β* = -.19), depression (*β* = -.50), and anxiety (*β* = -.19) were negatively associated, whereas work ability (*β* = .14) showed a significant positive association.

## Discussion

Our results revealed significant associations between education, depression, anxiety, and work ability with the MCS, and between age, gender, work ability, somatization, and anxiety with the PCS. No significant associations were found between cohabitation status, duration of unemployment, or social network size and either component of HRQoL.

The fact that our participants showed a lower MCS (*M* = 30.9, *SD* = 9.1) compared to the PCS score (*M* = 43.3, *SD* = 10.5) is in line with the findings from previous studies [2, 18]. A higher PCS compared to MCS may be indicative of slightly better physical functionality and a fewer everyday restrictions [18], whereas a lower MCS suggests reduced well-being and social participation [12]. In contrast to our results, similar research with unemployed individuals reported higher HRQoL scores, as in Italy (PCS: *M* = 49.0; MCS: *M* = 39.7) [2] and Spain (PCS: *M* = 46,3; MCS: *M* = 51,4) [32]. Similarly, a German study found a higher MCS (*M* = 44.0) and a marginally higher PCS (*M* = 44.6) in a comparable sample [3]. While PCS differences were not significant (*t*(815) = -1.82, *p* = .070, *d* = -0.13), our sample had substantially lower MCS scores (*t*(815) = -17.98, *p* < .001, *d* = -1.28), likely reflecting the combined burden of unemployment and mental illness.

Moreover, baseline data collection occurred from September 2020 until November 2023, partly during the COVID-19 pandemic, a period marked by increased psychological distress, potentially further reducing PCS and MCS. The implementation of lockdowns and other measures worldwide resulted in an initial surge in anxiety and depression symptoms [8, 33]. Pandemic-related stressors likely intensified existing vulnerabilities in this already high-risk group and could explain why the MCS and PCS are comparatively low.

Consistent with previous research, age was negatively associated with PCS, likely due to the natural decline in physical health with age [2]. No significant age effect was found for MCS. Likewise no gender difference was observed for MCS, but male gender was positively associated with PCS, possibly reflecting gender-based differences in health perception [18, 34].

Interestingly, a higher education levels was negatively associated with MCS, contradicting prior findings suggesting a protective effect [5]. This may be due to increased psychological strain from unmet professional expectations or loss of role identity after job loss [35]. Cohabitation status and social network (LSNS) size were not significantly related to HRQoL, contrasting previous studies showing protective effects [13, 36]. Psychiatric illness might diminish social support benefits through stigma-related experiences [37]. Moreover COVID-19-related contact restrictions may have masked this effect, as the functional relevance of social network size is reduced when interpersonal contact is limited [8]. Similarly, unemployment duration was unrelated to PCS and MCS in our sample. Unlike a German study reporting reduced PCS with very long-term unemployment (> 5 years) [3], our participants had shorter unemployment durations, suggesting that longer exposure may be needed to observe effects.

Furthermore, the self-assessed Work Ability Score (WAS) was positively associated with both MCS and PCS, indicating its role as a personal resource and a potential target for reintegration interventions [16, 38]. Work ability in our study reflects individuals’ perceived physical and mental capacity to work and can be understood as a form of work-related self-confidence. Given the likely bidirectional relationship between HRQoL and work ability [26], strengthening perceived work capacity may contribute to improvements in HRQoL.

The mean depression score (Mini-SCL) in our sample (10.32) represented the highest average value among all Mini-SCL domains and was markedly higher than the German population average (1.8) [39]. Its strong negative association with MCS highlights the relevance of depressive symptoms in this group. As job loss is a major stressor, unemployment may intensify depression [11]. Consistent with existing literature, the bidirectional relationship must be considered: while unemployment may exacerbate depressive symptoms, depression can make it difficult to re-enter the labor market, especially for individuals with limited social support [36, 40]. Early detection and treatment of depressive disorders in unemployed individuals is, therefore, essential.

Despite conceptual overlaps between depression and HRQoL [41, 42], considering depression improves the understanding of their combined effects [42], as it was linked to MCS, but not PCS in our data. This finding indicates that physical HRQoL is not significantly linked to depressive symptoms, suggesting that unemployed individuals with depression may still perceive their physical health as largely intact. PCS assesses self-reported physical functioning and is conceptually distinct from mental health constructs such as depression and anxiety. This maintenance of physical functioning despite depressive symptoms may reflect compensatory mechanisms, reduced occupational stress, or lower physical demands in those not currently employed [43]. These results have implications for the development of reintegration and return-to-work interventions, highlighting that physically demanding activities may be more tolerable than cognitively demanding tasks for this population [44].

The average anxiety score (Mini-SCL) in our sample (7.6) was notably higher than that reported in the German reference group (1.4) [39]. This may reflect the anxiogenic nature of unemployment, which is often accompanied by uncertainty about the future, financial insecurity, reliance on unstable benefits, and the stress of job seeking [9]. In our sample, anxiety was associated with lower MCS but slightly higher PCS scores, whereas previous research found elevated anxiety linked to poorer outcomes in both dimensions [3]. It is conceivable that those with higher levels of anxiety experience greater mental distress but are generally more cautious, utilizing healthcare services more frequently, and paying attention to good health resulting in higher PCS [45].

Somatization (Mini-SCL) in our sample (5.6) exceeded normative values (1.5) [27, 39] and were negatively associated with PCS but not MCS, aligning with the understanding that somatic symptoms may be perceived as physical limitations. The distinction between mental and physical health symptoms is not entirely clear-cut. Since mental illnesses have been demonstrated to be associated with physical symptoms, and conversely, physical illnesses have been shown to influence mental health, all relevant symptoms need to be addressed in reintegration efforts [46, 47]. At this point, it is crucial to distinguish between specific forms of psychological distress (depression, anxiety, somatization) and the broader MCS, which reflects general psychological well-being and functioning [48]. We can observe this using the following example items of Mini-SCL and SF-12. Some items of the Mini-SCL focus rather on the assessment of specific symptoms: *How much have you suffered during the past seven days from 3) nervousness or inner trembling (anxiety) or 7) nausea or stomach upset (somatization) or 14) a feeling of hopelessness regarding the future (depression)? Responses could be given on a scale from 0* = *“not at all” to 4* = *“very strongly.”*

By comparison, items of the SF-12 evaluate all symptoms concerning physical and mental health in relation to daily functioning reflecting quality of life, here MCS (Item 6 & 7): *During the past 4 weeks, have you had any of the following problems with your work or other regular daily activities as a result of any emotional problems (such as feeling depressed or anxious)? 6. Accomplished less than you like (yes/no) or 7. Didn’t do work or other activities as carefully as usual (yes/no).*

Clinicians and professionals should consider job loss as a risk factor for physical and mental health detoriation, especially in those with pre-existing mental health symptoms [34]. Including HRQoL as a concise self-assessment tool may aid in monitoring high-risk groups over time. Our findings suggest that unemployed people with mental illnesses constitute a vulnerable group in need of support to strengthen their resources. Interventions should aim to reduce symptoms of depression, anxiety and somatization in this high-risk group and to prevent existing symptoms from worsening [9]. Cognitive behavioral therapies [16], physical activity [49], work-related measures (such as Individual Placement and Support) [50, 51] are supported by evidence. From an HRQoL perspective, psychosocial approaches [18] and strengthening subjective health competence [52] may be beneficial.

To more accurately identify areas requiring intervention and to develop effective measures, future research may focus on specific subgroups, such as single parents [53] or young individuals struggling with addiction [54]. One potential limitation of this study is that the data were collected during the course of the COVID-19 pandemic, which could have resulted in data bias, as was considered during interpretation. The present analyses are limited by the stringent exclusion of cases with missing data as well as by the use of dichotomous variables. Another limitation is that the study design is cross-sectional, which complicates the directional interpretation of effects. A longitudinal study could be conducted in the future to investigate the long-term relationships and causality between unemployment, mental health and HRQoL.

## Conclusion

The present study has demonstrated associations between mental and physical dimensions of health-related quality of life and several factors, including age, gender, level of education, work ability, depression and anxiety, in a group of unemployed individuals with mental illnesses. Our findings provide important implications for diagnostic, preventive, and therapeutic support planning for this vulnerable group.

## Data Availability

The data that support the findings of this study are available in anonymized form from the corresponding author upon reasonable request.
